# Neutralizing and non-neutralizing antibody responses in HIV-1 subtype C chronically infected patients with divergent rates of disease progression

**DOI:** 10.1186/1742-4690-9-S2-P75

**Published:** 2012-09-13

**Authors:** D Archary, R Rong, ML Gordon, S Boliar, ES Gray, A Dugast, T Hermanus, PJ Goulder, HM Coovadia, L Morris, G Alter, CA Derdeyn, T Ndung'u

**Affiliations:** 1 University of KwaZulu-Natal, Durban, South Africa; 2 Jiaotong-Liverpool University , Suzhou, China, China; 3 Emory University, Atlanta, GA, USA; 4 University of Western Australia, Australia; 5 Ragon Institute, Boston, MA, USA; 6 National Institute for Communicable Diseases, Johannesburg, South Africa; 7 Oxford University, UK; 8 University of KwaZulu Natal , Durban, South Africa

## Background

Development of an efficacious HIV-1 vaccine able to elicit the production of broadly neutralizing antibodies (nAbs), capable of retaining potent activity against a diverse panel of viral isolates remains a significant challenge. The evolutionary forces that shape envelope and ensuing nAb and non-neutralizing antibodies in HIV-1 subtype C are incompletely understood and these two parameters have been rarely studied concurrently.

## Methods

We characterized patterns of virus-specific nAbs and non-neutralizing antibodies in four slow progressors and four progressors with chronic HIV-1 subtype C infection, over a median of 21 months. Single cycle neutralization assays was performed. In addition, the binding affinities of HIV-specific immunoglobulins (IgGs) and the affinities of the IgGs to various Fcγ receptors (FcγRs) were assessed.

## Results

NAbs evolved significantly in progressors (p=0.003) from study entry to study exit. NAb IC50 titers significantly correlated with amino acid lengths for V1-V2 (p=0.04), C3-V5 (p=0.03) and V1-V5 (p=0.04). Both groups displayed preferential heterologous activity against the subtype C panel. Both groups displayed preferential heterologous activity against the subtype C panel. There were no significant differences in breadth of responses between the groups for either subtype A or C. Neutralization breadth and titers to subtype B reference strains was significantly higher in progressors compared to slow progressors (both p<0.03) with increasing nAb breadth from study entry to study exit in progressors. Progressors had cross-reactive neutralizing antibodies that targeted V2 and V3. Binding affinities of non-neutralizing antibodies to HIV-specific gp120, gp41 and p24 and to activating and inhibitory Fcγ receptors (FcγRs) were similar in both groups. However, in slow progressors, CD4 T-cell counts correlated inversely with antibody binding affinity for the activating FcγRIIa (p=0.005).

## Conclusion

Overall, the data suggest that neither nAbs nor non-neutralizing antibodies could be directly associated with disease attenuation. However, continuous evolution of nAbs was a potential marker of disease progression.

